# Evolution of Duplicated Glutathione Metabolic Pathway in *Gossypium hirsutum* and Its Response to UV‐B Stress

**DOI:** 10.1002/ece3.70537

**Published:** 2024-11-19

**Authors:** Xiaolin Song, Xiaoyu Yin, Yingjie Zhu, Qi Su, Ying Bao

**Affiliations:** ^1^ School of Life Sciences Qufu Normal University Qufu Shandong China

**Keywords:** co‐expression, GSH, regulatory network, UV‐B

## Abstract

Increasing levels of UV‐B radiation caused by the greenhouse effect has become an emerging threat to crop health and yield. The glutathione (GSH) metabolic pathway is generally involved in plant stress responses through scavenging accumulated reactive oxygen species, and is therefore believed to play an essential role in enhancing plant tolerance to UV‐B stress. However, the complex evolutionary details of this pathway in polyploid plants, especially under UV‐B stress, remain largely unknown. Here, using the important allotetraploid crop, *Gossypium hirsutum*, as an example, we comprehensively investigated the composition and phylogenetic relationships of genes encoding 12 key structural enzymes in this pathway, and compared the expression changes of all the relevant genes under UV‐B stress (16 kJ m^−2^ d^−1^) based on six leaf transcriptomes. Consequently, we identified 205 structural genes by genome‐wide searching and predicted 98 potential regulatory genes under multiple stress conditions by co‐expression network analysis. Furthermore, we revealed that 19 structural genes including 5 homoeologous pairs and 96 regulatory genes possessing 25 homoeologous pairs were reticulately correlated without homoeologous selection preference under UV‐B stress. This result suggests a complex rewiring and reassignment between structural genes and their regulatory networks in the duplicated metabolic pathways of polyploid cotton. This study extends our understanding of the molecular dynamics of the GSH metabolic pathway in response to UV‐B stress in *G. hirsutum* and, more broadly, in polyploid plants.

## Graphical abstract

Differential and reassigned expression of duplicated structural genes and regulatory networks in the GSH metabolic pathway under UV‐B duress..

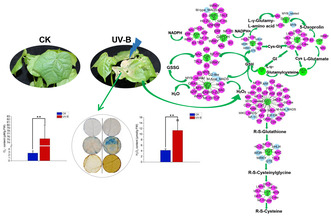

## 1 Introduction

Ultraviolet‐B (UV‐B, 280–315 nm), a component of the solar electromagnetic spectrum that reaches the Earth's surface, has received widespread attention in recent years because anthropogenic carbon dioxide emissions are destroying the ozone layer, resulting in increasing UV‐B radiation (Liaqat et al. 2023). UV‐B irradiation can cause significant alterations in plants morphology, physiological characteristics, and ultrastructure, leading to a reduction in plants biomass and yield (Kakani et al. 2003; Liaqat et al. 2023). At high flux rates, UV‐B usually acts as a damaging agent causing harmfulness to plants biomolecules by generating excess reactive oxygen species (ROS). ROS comprising the singlet oxygen (^1^O_2_), superoxide (O_2_^•−^), hydrogen peroxide (H_2_O_2_), and hydroxyl radicals (•OH) can trigger oxidation of lipid and protein, damage DNA and RNA, and reduce cell viability (Shi and Liu 2021). In response to increased ROS, plants have developed various effective defense mechanisms by different antioxidants (Mahmood et al. 2019). Some antioxidants such as ascorbic acid, carotenoid, flavonoids, and phenolic have been proved to be effective to defense oxidative stress induced by UV‐B radiation (Dias et al. 2020; Xie et al. 2022). These antioxidants are synthesized in several metabolic pathways. The gene composition, expression patterns and regulatory networks of the pathways under UV‐B stress in plants are largely unknown.

Glutathione (γ‐glutamyl‐cysteinyl‐glycine, GSH), a tripetide of cysteine, glutamic acid, and glycine, is an important antioxidant, radical scavenger, and antidote, which is essential for protecting cell, preserving enzymes activities, and proteins functions, preventing cytoplasmic and outer membranes damage (Hasanuzzaman et al. 2019). GSH and glutathione S‐transferase (GST) mediated antioxidant processes were enhanced after UV‐B treatment (Yang et al. 2007). The GSH metabolic pathway is incorporated with multiple precursors and intermediates. GSH is synthesized from the amino acid substrates glutamate, cysteine (Cys), and glycine (Gly) by the consecutive reactions of glutamate cysteine ligase (GCL) and glutathione synthetase (GS) (Dorion, Ouellet, and Rivoal 2021). In addition, a γ‐glutamyl transpeptidase (GGT) can maintain GSH homeostasis by breaking down extracellular GSH and providing Cys, and promote intracellular de novo synthesis of GSH. The γ‐glutamyl cyclotransferase (GGCT) catalyzes the formation of 5‐oxoproline from γ‐glutamyl dipeptides, and with the help of 5‐oxoprolinase (OXP) to generate glutamate (Dorion, Ouellet, and Rivoal 2021). GGT can also initiate the metabolism of glutathione S‐conjugates to mercapturic acids by transferring the γ‐glutamyl moiety to an acceptor amino acid and releasing cysteinylglycine (Cys‐Gly). Cyc‐Gly is then broken down by leucine aminopeptidases (LAP) to generate Cys or Gly, and Cys and Gly recycled to the process of GSH synthesis (Kumar et al. 2015). A study in *Arabidopsis thaliana* revealed that oxidative stress conditions caused by UV‐B irradiation and disruption of the γ‐glutamyl cycle resulted in a similar stress‐induced response (Masi, Trentin, and Arrigoni 2016). GSH is then oxidized during peroxide disposal by glutathione peroxidases (GPX) or dehydroascorbate reductase (DHAR) to GSSG (oxidized form of GSH) and is regenerated by glutathione reductase (GR) from GSSG at the expense of NADPH (Dorion, Ouellet, and Rivoal 2021). Higher GSH content under stress is considered to maintain GSH/GSSG ratio. In this process, the orchestrated action of antioxidant enzymes, GPX and GR, are able to control the cellular concentration of O_2_^•−^ and H_2_O_2_, thereby preventing the formation of reactive radicals (Dorion, Ouellet, and Rivoal 2021). Glucose‐6‐phosphate dehydrogenase (G6PDH) and 6‐phosphogluconate dehydrogenase (PGDC) yield NADPH, a crucial cofactor of the enzyme GR converting GSSG into GSH (Cui et al. 2023). Some authors thought that elevated G6PDH activity played a key role in the control of GSH levels by utilizing NADPH under UV‐B stress (Dorion, Ouellet, and Rivoal 2021; Cui et al. 2023). NADPH can be synthesized by isocitrate dehydrogenase (IDH) (Moreno‐Sanchez et al. 2018). GSH can also react with xenobiotics and endogenous compounds in reactions catalyzed by GST. Obviously, the GSH metabolic pathway including diverse structural enzymes is a very complicated process. Especially, several key structural enzymes in the pathway are encoded by multiple gene families, which undoubtedly adds complexity to the pathway. Furthermore, there is little information about the regulatory network for this pathway. Therefore, although the GSH pathway has been found to be associated with UV‐B defense, the detailed characteristics of the structural and regulatory system of this pathway require further comprehensive study.

*Gossypium* L. is an economically important genus for providing natural fiber and seed nutrients that are widely used in textiles, industry, medicine, food, and feed. A total of four species are domesticated in the genus, including two AA genome diploids (*G. arboreum* L. and *G. herbaceum* L.) and two AADD genome allotetraploids (*G. barbadense* L. and *G. hirsutum* L.). However, due to the high yield and wide adaptability, *G. hirsutum*, that is, upland cotton, has been the major cotton species grown globally and accounts for more than 90% of the production share in the international cotton fiber market (Wendel, Flagel, and Adams 2012). Therefore, *G. hirsutum* received more attention than the other three domesticated cottons in agriculture. In addition, this species, together with *G. barbadense*, can also provide a model system for studying the complex evolution of polyploid crops. *G. hirsutum* originated in approximately 1–2 million years ago by hybridization of two diploid species with AA and DD genomes and concomitant polyploidization (Wendel, Flagel, and Adams 2012; Peng et al. 2022). As an allotetraploid species, upland cotton combines two sets of parental homoeologous genes (A‐ and D‐ subgenomic homoeologs, hereafter abbreviated as At‐homoeolog and Dt‐homoeolog, where *t* represents tetraploid) into a common nucleus, and thus possesses duplicated homoeologous pairs of both structural and regulatory genes in any metabolic pathway in most cases. This situation provides additional opportunities to rearrange or assign these duplicated homoeologous genes in the original parallel pathways, leading to neofunctionalization, subfunctionalization, non‐functionalization, and complete loss of either or both of the diploid parental homoeologous genes (Chaudhary et al. 2009; Wendel, Flagel, and Adams 2012). Obviously, clarifying the coexistence characteristics of these duplicated genes in the same pathway in *G. hirsutum* will provide more insights to further reveal the evolutionary patterns of other polyploid crops and enhance the effectiveness of molecular breeding in cotton.

With the changes in stratospheric ozone and climate over the past years (Liaqat et al. 2023), crops, including *G. hirsutum*, inevitably face an increasingly pronounced threat of UV‐B radiation. Given that the consistent role of the GSH metabolism in antioxidants, in this study we sought to unravel the molecular basis of the pathway during UV‐B stress resistance in upland cotton, and mainly attempt to explore four questions: (1) How many genes or gene members are in different structural enzyme families of the GSH pathway? (2) Which structural genes or members are differential expressed in the pathway during UV‐B stress? (3) What regulatory genes might be involved in the GSH pathway, and who might regulate the UV‐B resistance process? (4) What are the evolutionary characteristics of the duplicated GSH pathway in polyploid cotton?

## 2 Materials and Methods

### 2.1 Plant Materials and Growth Conditions

Experiments were conducted in a controlled environment of the greenhouse in Qufu Normal University, China. Seeds of an elite cultivar *G. hirsutum* cv. Acala Maxxa, kindly provided by Wendel laboratory in Iowa State University, were sown in plastic pots (10 × 10 cm) containing a mixture of nutrient soil and vermiculite in a ratio of 3:1. From sowing to three‐leaf stage, plants were grown in a growth chamber with a photoperiod of 16 h of light and 8 h of darkness at temperature of 23°C ± 1°C.

An earlier study confirmed that cotton could produce a stress response when exposed to high UV‐B level (16 kJ m^−2^ d^−1^) (Kakani et al. 2003). Here, to examine the response of cotton seedlings to UV‐B stress, each three plants were placed under normal control condition (CK) or 6 h of UV‐B radiation (16 kJ m^−2^ d^−1^). A UV‐B lamp (Beijing Zhongyi Boteng Technology Co. Ltd.) was used to generate UV‐B radiation. After treatment, the second and third true leaves of control and treated plants were collected for physiological index determination, RNA extraction, transcriptome sequencing, and gene expression quantitative comparison. All the treatments were conducted in triplicates.

### 2.2 Histochemical Detection of the Content of O_2_^•−^, H_2_O_2_ and the Status of Cell Damage

The histological detection of O_2_^•−^ and H_2_O_2_ in cotton leaves by using NBT (Nitroblue tetrazolium chloride, Beijing Boaotuo Technology Co. Ltd.) and DAB (DAB 4HCL, Beijing Boaotuo Technology Co. Ltd.) as the chromogenic substrate as described by Lee et al. (2023), respectively. Well‐grown leaves of CK and UV‐B treated cotton seedlings were selected, and leaf discs of 1 cm diameter were punched out with a hole puncher. These leaf discs were then immersed with 5 mL NBT solution (0.5 mg/mL) or DAB solution (1 mg/mL), wrapped with foil and incubated for 12 h at room temperature. After incubation, the stained leaf discs were bleached in 90% ethanol at 70°C, and then stored in 50% glycerol until photographic documentation was completed. In leaves, NBT reacts with O_2_^•−^ to form dark blue insoluble formazan compound, while DAB is oxidized by H_2_O_2_ in the presence of peroxidases to produce reddish brown precipitate. In addition, the content of O_2_^•−^ in leaves was determined using the method of Elstner and Heupel (1976). A hydrogen peroxide kit (Cat. H_2_O_2_‐1‐Y, Suzhou Comin Biotechnology Co. Led, Jiangsu, China) was used to detect the content of H_2_O_2_.

Cell damage is characterized by membrane disruption, and the Evan's blue stain can penetrate through the destabilized membrane and stain the cells (You et al. 2021). Here, Evan's blue stain was used to evaluate the membrane damage of upland cotton seedlings under UV‐B stress. Leaf discs were stained with Evans blue solution (0.5%) (Shanghai Maokang Biotechnology Co. Ltd., China) for 12 h in the dark, then washed three times with distilled water and immersed in boiling anhydrous ethanol‐glycerol (9:1 v/v) solution for 30 min to remove chlorophyll.

### 2.3 Identification and Analysis of Structural Genes in the GSH Metabolic Pathway

Based on previous studies (Moreno‐Sanchez et al. 2018; Dorion, Ouellet, and Rivoal 2021; Cui et al. 2023), we selected 12 key structural enzymes (G6PDH, GCL, GGCT, GGT, GPX, GR, GS, GST, IDH, LAP, OXP, and PGDC) in the GSH metabolic pathway, all of which are encoded by gene families. We then scanned and downloaded all CDS and protein sequences encoding the 12 structural enzymes in *G. hirsutum* using the sequence similarity blasting and keyword searching on the CottonFGD website (https://cottonfgd.org/). Given that the *GST* gene family is the largest and most complex family in the GSH pathway, in order to comprehensively identify all gene members in the family, we used the hidden Markov model (HMM) profile of *GST* (GST_N, PF00043; GST_C, PF02798) from Pfam (http://pfam.xfam.org/) as the query entry to perform a further BLAST search against the protein data of the *G. hirsutum* genomes with a threshold of E‐value ≤ 1e^−5^. Different search results were final merged and redundant genes were removed. Meanwhile, the protein sequences of the retrieved genes were further verified using the Conserved Domain Search (https://www.ncbi.nlm.nih.gov/cdd/), SMART (http://smart.embl‐heidelberg.de/), and compared with the annotated proteins in the Pfam database (http://pfam.xfam.org).

The CDS and protein lengths of all structural genes were analyzed using TBtools (v. 2.083) software (Chen et al. 2023). Protein sequences of 12 gene families were aligned using ClustalW program in MEGA (v.11) software (Tamura, Stecher, and Kumar 2021). Pairwise sequence similarity for each gene family were calculated using the MatGAT software (Campanella, Bitincka, and Smalley 2003). The number of introns and exons of the genes was summarized using the exon‐intron structure function on the CottonFGD website.

### 2.4 Phylogenetic Tree Construction

In order to explore the phylogenetic relationships of the genes encoding 12 structural enzymes in the GSH pathway of *G. hirsutum*, a maximum likelihood (ML) tree was constructed based on the amino acid sequences of these genes under the Jones‐Taylor‐Thornton (JTT) model with 500 bootstrap replications using the MEGA (v. 11) software. FigTree v1.4.4 (http://tree.bio.ed.ac.uk/software/figtree/) and Adobe Illustrator 2022 was used to visualize and edit the ML tree, respectively.

### 2.5 RNA Extraction and Transcriptome Sequencing

Total RNAs of leaves in the CK and UV‐B stress treatment (UV) groups were extracted using RNAprep Pure Plant Kits (Cat. No 4992237, TIANGEN, Beijing, China) following the manufacturer's instructions, respectively. RNA integrities and quantities were assessed using the RNA Nano 6000 Assay Kit on the Bioanalyzer 2100 system (Agilent Technologies, CA, USA).

Six cDNA sequencing libraries (each three replicates of CK and UV) were constructed using NEB Next Ultra RNA Library Prep Kit for Illumina (New England Biolabs, MA, USA) following the manufacturer's recommendations. The library fragments were purified using the AMPure XP system (Beckman Coulter, Beverly, USA) and sequenced on an Illumina NovaSeq 6000 platform. Library construction and transcriptome sequencing were achieved by Beijing Novogene Bioinformatics Technology Co. Ltd.

### 2.6 Gene Co‐Expression Network Construction and Visualization

In order to fully understand the regulatory networks of the GSH pathway under UV‐B stress, we employed the co‐expression network analysis to search for potential regulatory genes of different structural genes. Since co‐expression analyses require at least 15 samples to ensure the robustness and repeatability of the results. Therefore, in addition to the current three UV‐B stress transcriptomes, we included additional 30 published transcriptome data of *G. hirsutum* under other stress treatments (drought, salt, heat, cold, CdCl_2_, NaOH, NaHCO_3_, and Na_2_SO_4_) in subsequent analyses. The 30 transcriptome data were downloaded from the NCBI website (https://www.ncbi.nlm.nih.gov/Traces/study/). The SRA files of a total of 33 transcriptomes (Table S1) were converted into fasta files by using the fastq‐dump (v.2.8.0) function of the SRA Toolkit (v.2.9.4) software (http://www.ncbi.nlm.nih.gov/books/NBK158900/). Raw data of the RNA‐sequences were cleaned using Trim Galore (v. 0.6.10) software (Wang et al. 2022). Gene expression was quantified by salmon (v. 0.13.1) method (Patro et al. 2017) after indexed according to cotton reference genome (UTX_v2.1) (Chen et al. 2020). The resulting read count tables were normalized by *rlog* transformation, built in DESeq2 (Love, Huber, and Anders 2014). Gene co‐expression networks were constructed using the WGCNA package in the R software (Langfelder and Horvath 2008). All data were clustered to analyze the sample height. The soft threshold was chosen according to *R*squared > 0.85. The topological overlap matrix (TOM) was created using the resultant adjacency matrix. The genes were hierarchically clustered by TOM similarity, and the hierarchical clustering tree was cut by dynamic hybrid tree cutting algorithm to obtain modules. In order to comprehensively analyze the co‐expression relationships between structural and regulatory genes, the cut‐off value of weighted edge obtained from the WGCNA was set at a low value of 0.2.

The structural genes were used as target genes to search for their potential regulatory genes in the pathway based on the weighted co‐expression correlations. Several co‐expression networks were integrally generated based on cut‐off weight values, and were visualized using Cytoscape software (v.3.8.0) (Shannon et al. 2003).

### 2.7 Detection of Differentially Expressed Genes (DEGs) in the GSH Pathway Under UV‐B Stress

In order to detect the DEGs of the GSH pathway under UV‐B stress, we assembled, relatively quantified, and annotated all genes in six leaf transcriptomes of the CK and UV groups. Paired‐end clean reads were aligned to the cotton reference genome (UTX_v2.1) (Chen et al. 2020) using Hisat2 (v2.0.5) (Mortazavi et al. 2008). The mapped reads for each sample were assembled using StringTie (Pertea et al. 2015). The DEseq2 package (Love, Huber, and Anders 2014) was used to perform a differential expression analysis of the genes between the UV and CK groups (three biological replicates per group). The *p‐*value for each gene was adjusted (*padj*) using the Benjamini and Hochberg's method (Benjamini and Hochberg 1995). Genes with the adjusted *padj* < 0.05 and absolute log_2_ fold changes (log_2_FC) > 1 were considered as DEGs. Due to the influence of sequencing depth and gene length, the gene expression value of RNA‐seq in subsequent analysis was represented by FPKM (fragments per kilobase of transcript per million reads mapped).

### 2.8 Functional Enrichment of DEGs

Gene ontology (GO) term clustering and Kyoto Encyclopedia of genes and genomes (KEGG) pathways enrichment analysis of the DEGs was implemented by the clusterProfiler R package (Yu et al. 2012), in which gene length bias was corrected. The GO terms and KEGG pathway with corrected *padj* < 0.05 were considered significantly enriched in the DEGs. Based on the results of GO and KEGG enrichment analysis, the genes that were differentially expressed in the GSH metabolic pathway under UV‐B treatment were identified. The online website ImageGP (https://www.bic.ac.cn/ImageGP/) was used to draw a heatmap of DEGs using normalized expression values of log_2_(FPKM+1).

### 2.9 Expression Level Validation of the DEGs by Quantitative Real‐Time PCR (qRT‐PCR)

To verify the expression levels of the DEGs, qRT‐PCR amplifications of 17 randomly selected genes were performed on a LightCycler 480 II instrument (Roche Diagnostics GmbH, Germany) using the RNAs as for the library construction and illumina sequencing. Gene *PU1* (*Gohir.A01G131900*) was used as internal control. The primers of these genes for the qRT‐PCR analysis were designed and listed in Table S2. Relative expression of these genes were calculated according to the 2^−ΔΔCT^ method (Schmittgen and Livak 2008). *T*‐test was applied for significant differences among treatments at *p* < 0.05 level. The results were shown as the means ± standard error of three replicates.

### 2.10 Relative Expression Changes of Homoeologous Genes Under UV‐B Stress

To detect the relative expression changes of homoeologous genes, the ratio of At‐homoeolog to Dt‐homoeolog (At/Dt) at expression level was calculated for each pair of genes. The ratio with no statistical significance (*p* ≥ 0.05) was recorded as 1. Then, relative expression status of each homoeologous pair was classified into unbiased (At/Dt = 1) and biased (At/Dt ≠ 1) expression. Unbiased expression refers to the absence of expression bias between At‐ and Dt‐ homoeologs under CK and UV‐B stress conditions (no bias) or only under UV‐B stress (lost bias); biased expression refers to the existence of expression bias between At‐ and Dt‐ homoeologs under UV‐B stress. For the latter case, which can further be classified into four situations: (1) no expression bias occurred under CK condition, but the expression of At‐homoeolog dominated under UV‐B stress (Get At bias); (2) no expression bias occurred under CK condition, but the expression of Dt‐homoeolog dominated under UV‐B stress (get Dt bias); (3) significant expression bias occurred between At‐ and Dt‐ homoeologs under CK condition, but the expression of At‐homoeolog dominated under UV‐B stress (At bias); (4) significant expression bias occurred between At‐ and Dt‐ homoeologs under CK condition, but the expression of Dt‐homoeolog dominated under UV‐B stress (Dt bias).

## 3 Results

### 3.1 Effects of UV‐B Stress on the Leaves of *G. hirsutum*

**FIGURE 1:**
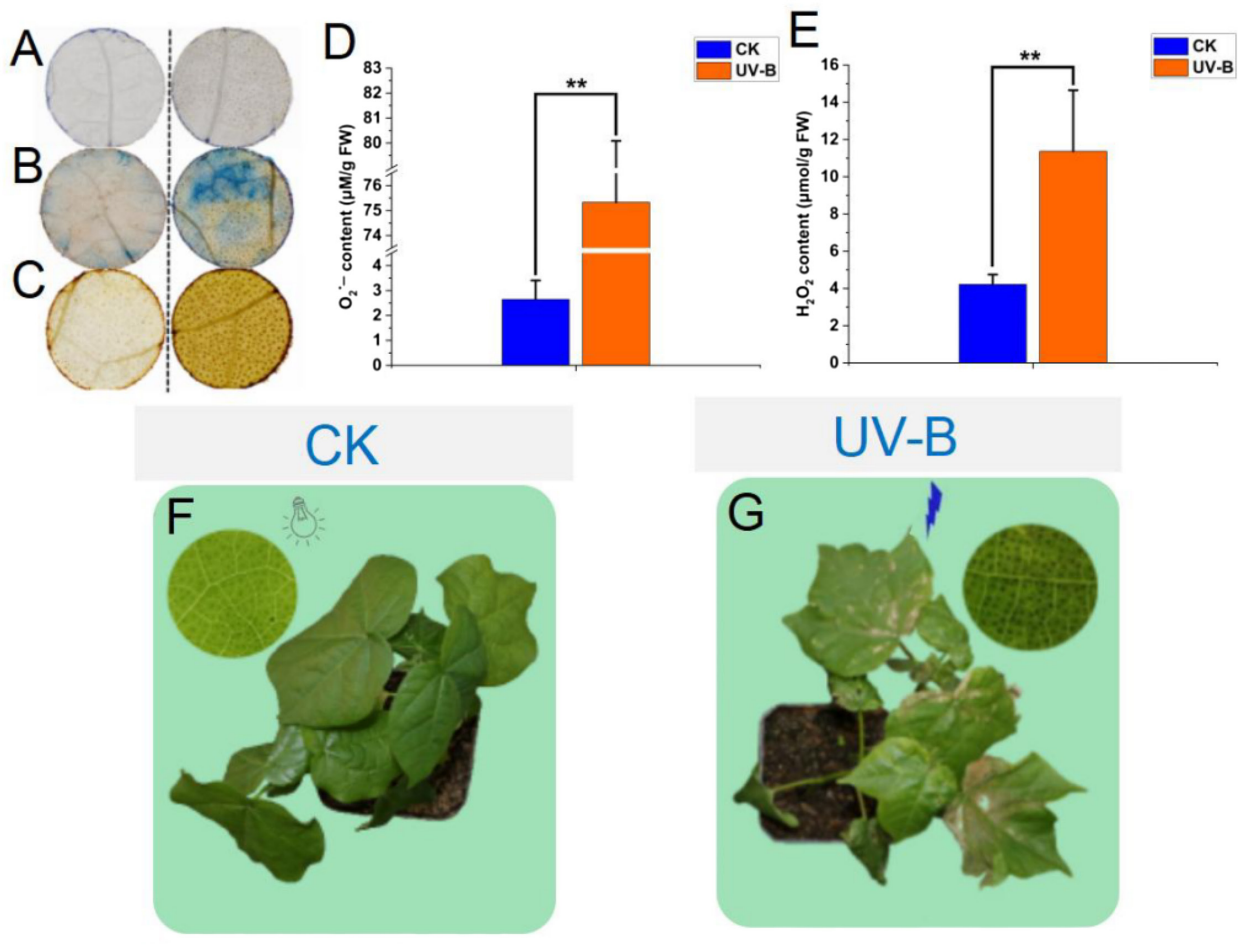
Stress treatment, histochemical staining, and physiological index analysis of *G. hirsutum*. (A–C) The NBT assay, Evans Blue stain, and DAB stain were used to visualize the content of O_2_^•−^ in leaves, cell damage, and the content of H_2_O_2_ in leaves. (D, E) The reaction of hydroxylamine and superoxide anion radicals and hydrogen peroxide kit were used to detect the content of O_2_^•−^ and H_2_O_2_ in leaves between CK and UV‐B group. (F, G), Plant phenotypes under natural condition for 6 h (CK) or UV‐B (16 kJ m^−2^ d^−1^) (UV‐B) for 6 h. Statistical significance was calculated using T‐test; *p < 0.05, **p < 0.01.

The effect of current UV‐B stress (16 kJ m^−2^ d^−1^) on cotton leaves induced significant physiological responses immediately after 6 h of treatment. Indicative signs, such as distinct brown spots (Figure 1A), dark blue stains (Figure 1B), and brown precipitation (Figure 1C), appeared in the staining of UV‐B‐treated leaves by NBT, Evans blue, and DAB, respectively. In addition, the levels of O_2_^•−^ and H_2_O_2_ in the leaves of the UV group were significantly increased by 28‐fold (Figure 1D) and nearly three‐fold (Figure 1E), respectively, compared with those of the CK group. Notably, phenotypic effects of UV‐B stress on cotton leaves were lagging. It was not until 2 days after UV‐B treatment that cotton leaves showed visible signs of drying and wilting (Figure 1F,G). These results suggest that UV‐B stress promotes the accumulation of O_2_^•−^ and H_2_O_2_ in cotton leaves, and the increased ROS content eventually damages leaf cells.

### 3.2 Structural Genes Evolution of the GSH Metabolic Pathway in *G. hirsutum*

After scanning the whole genome of *G. hirusum*, we identified a total of 205 structural genes in the GSH metabolic pathway (Table S3). Sequence analysis showed that most of the genes encoding 12 structural enzymes in the tetraploid cotton were doubled by hybridization and polyploidization. These 205 genes actually involve 125 gene loci, of which 80 include both At‐ and Dt‐homoeologs (160 genes), 18 and 27 have only At‐homoeologs or Dt‐homoeologs, respectively. With the exception of the GCL and GS, which are encoded by a pair of homoeologs genes, the other 10 enzymes (G6PDH, GGCT, GGT, GPX, GR, GST, IDH, LAP, OXP, and PGDC) in the cotton genome are encoded by multiple gene loci, ranging from two in the *OXP* families to 76 (121 genes) in the *GST* family. In addition, according to an earlier study on diploid cottons (Dong et al. 2016), these *GST* genes can further subdivided into eight classes, namely *GSTF* (*Phi*), *GSTU* (*Tau*), *GSTT* (*Theta*), *GST*Z (*Zeta*), *GSTL* (*Lambda*), *DHAR* (dehydroascorbate reductase), *TCHQD* (tetrachlorohydroquinone dehalogenase), and *EF1Bγ* (the eukaryotic translation elongation factor 1B). Here, we found out 70 *Tau*, 15 *Phi*, 10 *Theta*, seven *Lambda*, six *DHAR*, four *Zeta*, three *EF1Bγ*, and two *TCHQD*. Moreover, we also identified four additional genes (two loci) that have glutathione S‐transferases structural domains but encode microsomal glutathione S‐transferases (MGST) (Table S3).

These structural genes are distinctive in terms of gene length, gene structure, and sequence similarity. The shortest gene is a *GST* gene (*Gohir.D13G137000*) with a length of 261 bp, and the longest gene is an *OXP* gene (*Gohir.D02G190400*) with a length of 3873 bp. In addition, gene length polymorphisms varied across the 12 gene families. The sequence lengths of the gene members of the *GST* gene family differed the most, with an approximate five‐fold difference in gene length (261 bp vs. 1266 bp), whereas the sequence lengths of two genes (a pair of homoeologs) of the *GS* gene family were exactly equal (1650 bp). The gene structures of different members in the 12 gene families also varied, with a member (*Gohir.D02G122700*) in the *GR* family having the most exons (18), and three members (*Gohir.A11G134900*, *Gohir.D01G170400*, *Gohir.D11G141100*) in the *GST* family, two members (*Gohir.A03G167300*, *Gohir.D02G190400*) in the *OXP* family, and two members (*Gohir.A12G231700*, *Gohir.D12G235450*) in the *PGDC* family having the fewest exon (only one) (Table S3).

Pairwise comparison of protein sequences (Table S4) show that the sequence similarity profile within the 12 gene families is remarkably diverse. The greatest variation was in the *GST* gene family, with pairwise sequence similarity between ranging from 10.9% to 100%. Even within subfamilies, some gene pairs with low sequence similarity (< 30%) were identified in the *GSTF*, *GSTL*, *GSTT*, and *GSTU* classes of the *GST* gene family. Reanalyzing these divergent gene pairs, we found that when pairwise comparisons were made, one of the genes being too short in length would result in lower sequence similarity. For example, *Gohir.D08G200400* is the shortest gene (522 bp) in the *GSTT*s, the sequence similarity between this gene and the other longer gene *Gohir.D13G021400* (966 bp) in the *GSTLs* is only 24%. And a gene *Gohir.D04G134000* with the shortest length (309 bp) in the *LAP* gene family shared only 15%–18% sequence similarity with the other longer *LAP* genes (1530–1863 bp) (Tables S3 and S4). Notably, most of the short genes identified in this study are usually alternative splicing isoforms. The two genes (*Gohir.D04G134000* and *Gohir.D08G200400*) mentioned above have at least 10 and 3 isoforms, respectively. The result suggests that these particular genes may undergo different patterns of regulatory evolution.

**FIGURE 2:**
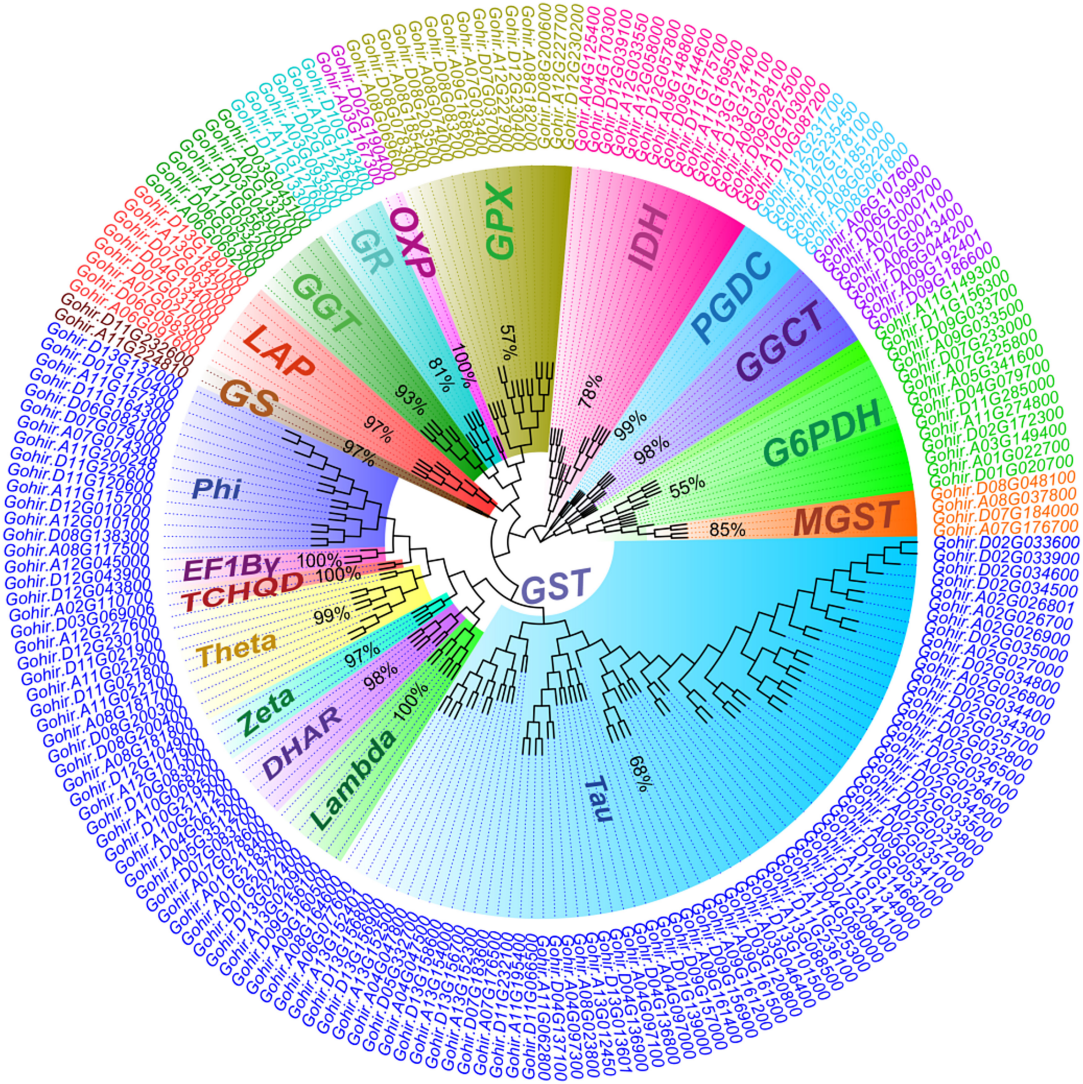
A ML tree of 12 structural gene families of *G. hirsutum*. The tree was constructed based on the amino acid sequences of 205 structural genes with 500 bootstrap replicates. Different family or subfamily clades are shadowed by different colors.

In order to evaluate the phylogenetic relationships of these 205 structural genes, we constructed a ML tree based on the amino acid sequences of these genes (Figure 2). Unsurprisingly, most of these structural genes encoding the same enzyme were grouped into the same clade in the tree, and most of gene loci were amplified by retaining homoeologous copies. In addition, eight subclades representing *DHAR*, *EF1Bγ, Lambda*, *Phi*, *Tau*, *TCHQD, Theta*, and *Zeta*‐like *GST* genes appeared in the ML tree. Among the subclades, the *Tau* subclade had the highest number of genes. At the same time, we also found that four *MGST* genes did not fall into the clade of *GST* gene family, but clustered with those *G6PDH* genes. This topology implies that these genes may have evolutionary divergence from other *GST* genes.

### 3.3 Regulatory Genes Evolution of the GSH Metabolic Pathway in *G. hirsutum*

**FIGURE 3:**
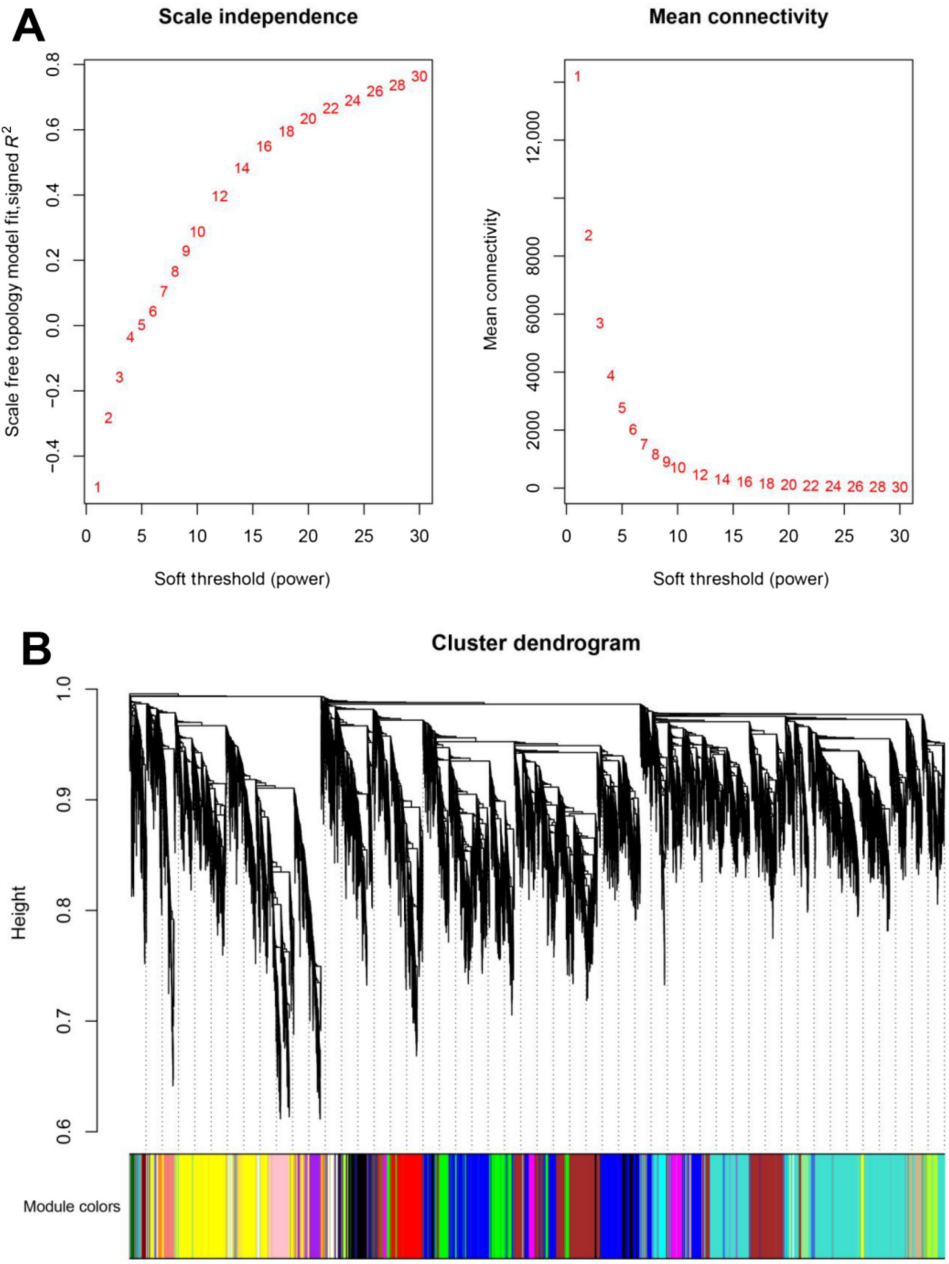
Soft‐threshold determination of gene co‐expression networks and module detection. (A) Determination of the soft threshold for dividing different modules. (B) Determination of the Hierarchical clustering tree. Each color represents a module in the co‐expression network by WGCNA.

**FIGURE 4:**
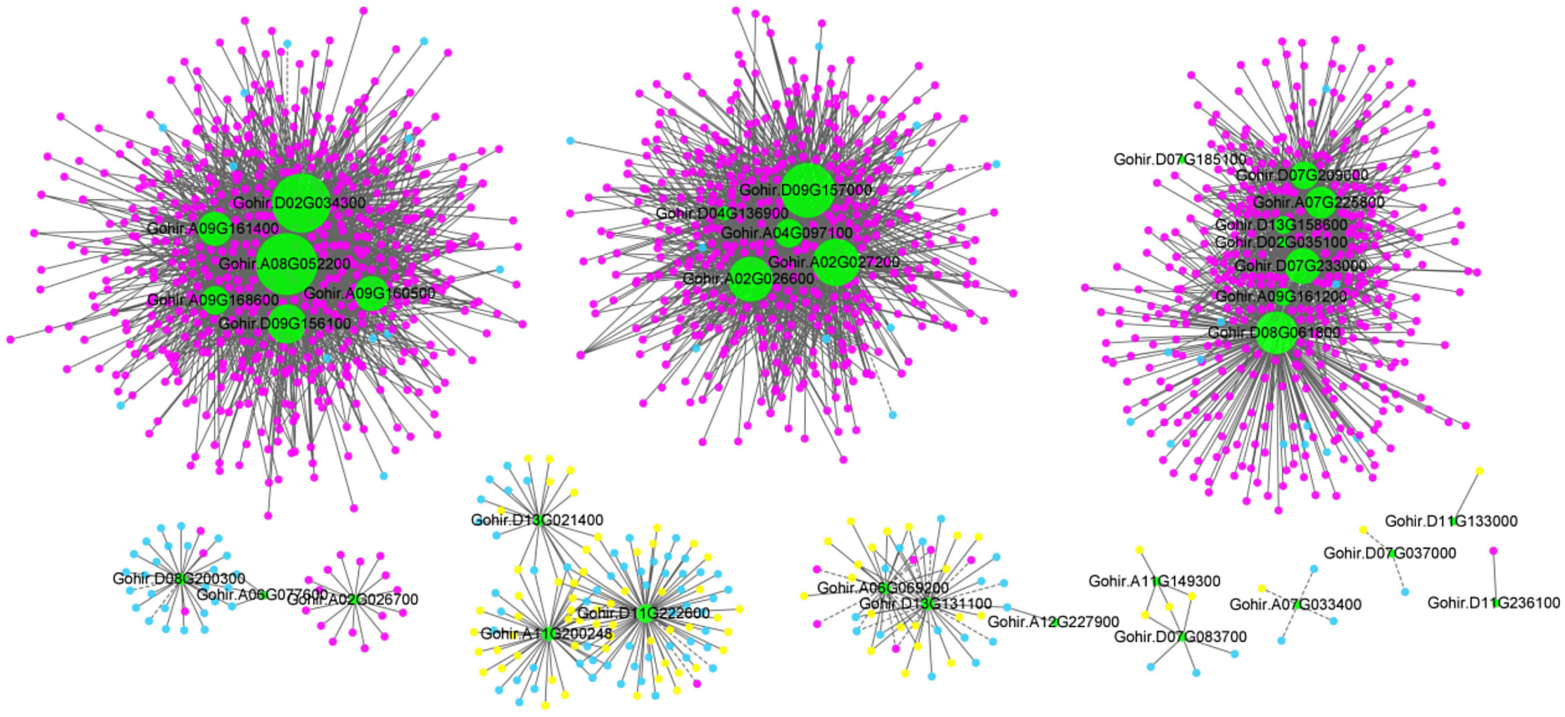
Correlation between structural genes and regulatory genes in different co‐expression networks. Green node: Structural gene; red node: Regulatory gene with up‐regulated expression under UV‐B stress; yellow node: Regulatory gene with down‐regulated expression under UV‐B stress; blue node: Regulatory gene with no significant difference in expression under UV‐B stress. Solid line: Positive regulation; dash line: Negative regulation.

**FIGURE 5:**
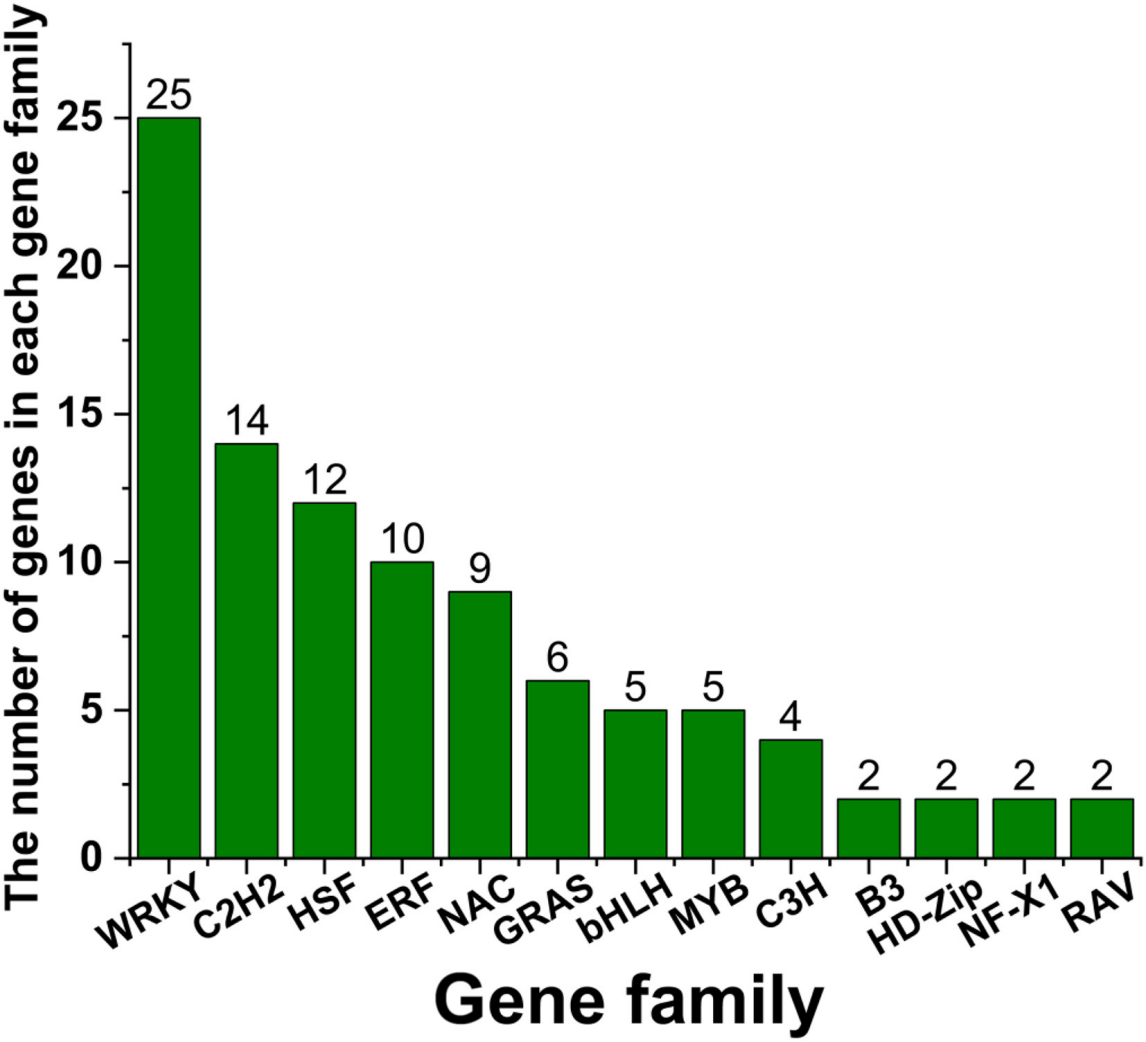
The distribution of gene numbers among different regulatory gene families identified from co‐expression networks. Horizontal axis: Name of the gene family. Vertical axis: Number of gene members per gene family.

In order to investigate the regulatory mechanisms of the duplicated GSH metabolic pathway in more detail, we constructed several co‐expression networks based on 33 stress‐related transcriptome data using the first 25,000 genes as targets (Figure 3A). Cluster analysis was performed on the 33 transcriptome data to obtain the correlation coefficients based on the expression level of each gene. After calculating the weight values, the genes with high correlation were assigned to the same module, and the modules with similar expression were merged by dynamic tree‐cut method (Figure 3B). Then, we used the structural genes in the GSH pathway as the target genes to screen out all potential regulatory genes from the co‐expression networks. Consequently, we found that under all stress conditions, only 21 structural genes (Table S5) appeared in these co‐expression networks, while 98 regulatory genes were predicated to be correlated with the 21 genes (Figure 4, Table S5). These regulatory genes were categorized into 13 gene families. Relatively abundant regulatory genes were identified from the *WRKY* (25), *C2H2* (14), *HSF* (12), and ERF (10) gene families. In contrast, only two regulators were detected in four (*B3*, *HD‐Zip*, *NF‐X1*, and *RAV*) gene families (Figure 5, Table S5).

Additionally, only three (*Gohir.A02G026700*, *Gohir.D02G035100*, and *Gohir.D13G131100*) of the 21 structural genes were correlated to specific regulatory genes, but the other 86% (18) shared at least one regulatory gene with other genes (Table S5). For example, a structural gene of *GSTU* (*Gohir.D02G035100*) was only correlated with a regulatory gene of *NAC017* (*Gohir.D02G067300*), whereas the other structural gene of *GSTU* (*Gohir.D13G158600*) was simultaneously correlated with three regulatory genes, *NAC017* (*Gohir.D02G067300*), *C2H2* (*Gohir.A11G051900*), and *WRKY28* (*Gohir.D10G039400*) (Figure 4, Table S5).

Among the 98 regulatory genes, we identified a total of 26 homoeologous pairs (52 genes) belonging to 13 gene families. Although the At‐ or Dt‐homoeolog in each pair encode the same enzyme, they were not always co‐expressed with the same set of target genes (Figure 4, Table S5). In some cases, At‐ or Dt‐homoeolog was correlated with different target genes in the same or different co‐expression networks and there was no subgenomic selection preference, that is, At‐ or Dt‐homoeolog don't only select genes with the same A or D subgenome for highly correlation, respectively. For example, the At‐homoeolog of a *WRKY24* gene (*Gohir.A03G025100*) was highly correlated with three structural genes, including a *GSTU* (*Gohir.D02G034300*), a *PGDC3* (*Gohir.A08G052200*), and a *GSTL3* (Gohir.A09G160500) genes. However, the Dt‐homoeolog of the *WRKY24* (*Gohir.D03G143400*) was correlated with only a *GSTU* gene (*Gohir.D02G034300*). In addition, co‐expression network analysis further showed that all but one gene (*Gohir.D07G048100*) of the 98 regulators positively regulated to their corresponding target structural genes (Table S5). These results indicate that the evolution of regulatory system for the duplicated GSH metabolic pathway in polyploid is complicated, reticulately correlated, with no independent parallel evolution of subgenomic components.

### 3.4 Expression Characteristics of the Structural Genes in the GSH Metabolism Pathway Under UV‐B Stress

In order to further elucidate the composition and express patterns of structural genes in the GSH metabolic pathway under UV‐B stress, we performed a RNA‐sequencing on cotton leaves under both UV‐B stress and control conditions. Six high‐quality transcriptomes (with mapping rates ≥ 94%, GC contents ≥ 43%, and Q30 values ≥ 93%), including approximately 41–45 million raw reads from each transcriptomes, were obtained (PRJNA893188). The clean reads ratio of the transcriptomes ranges from 98% to 99% (Table S6).

**FIGURE 6:**
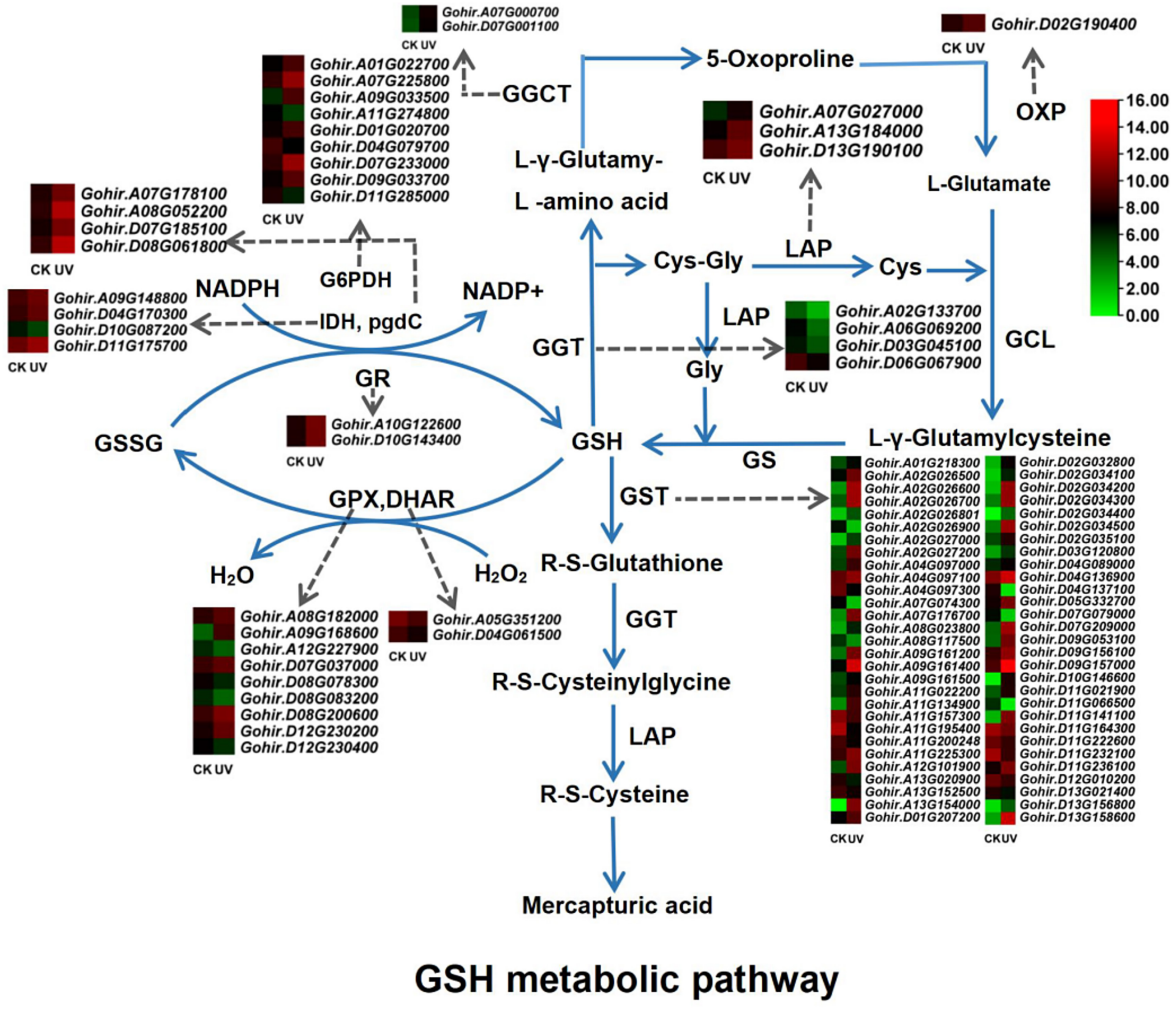
Pathway diagram and expression heat map of structural and regulatory genes related to the GSH metabolism. Pathway diagram shows 12 key enzymes in the GSH metabolic pathway; heat map shows structural genes with significantly differential expression under UV‐B stress.

Of the 205 structural genes characterized at the genome‐wide level, only 98 were DEGs under UV‐B stress (Figure 6, Table S3). In addition, these DEGs occurred in most structural gene families (*G6PDH*, *GGCT*, *GGT*, *GPX*, *GR*, *GST*, *IDH*, *LAP*, *OXP*, and *PGDC*) in the GSH metabolic pathway except for the *GCL* and *GS* families. The expression direction of these DEGs was not completely consistent, with 68 up‐regulated and 30 down‐regulated. Within gene families, two expression patterns have been identified. One pattern was that all gene members of the same family showed similar expression changes under UV‐B stress. For example, gene members in the *GGCT*, *GR*, *LAP*, *OXP*, and *PGDC* families were totally up‐regulated, whereas members in the *GGT* family were completely down‐regulated. Another pattern was that the expression of different gene members of the same family (*G6PDH*, *GPX*, *GST*, and *IDH*) was alternatively regulated in an upward or downward manner, respectively.

Furthermore, we found that only 19 of these 98 DEGs fell into the list of 21 structural genes in the co‐expression networks (Table S7). A *GSTU* gene, *Gohir.D02G034300*, was correlated with the largest number of regulatory genes (53). In contrast, two other *GSTU* genes, *Gohir.A02G026700* and *Gohir.D02G035100*, were correlated with a single regulatory gene each.

Among the 205 structural genes, 80 homoeologous pairs (160 genes) were identified (Table S3), of which, 72 pairs of homoeologs (144 genes) were simultaneously expressed under both CK and UV‐B stress conditions. The relative expression pattern of At‐ to Dt‐homoeolog of each homoeologous pair could be classified into unbiased (At/Dt = 1) and biased (At/Dt ≠ 1) expression. Unbiased expression included no bias (33 pairs, 66 genes) and lost bias (14 pairs, 28 genes); and biased expression included four patterns of At bias (six pairs, 12 genes), Dt bias (nine pairs, 18 genes), get At bias (six pairs, 12 genes), and get Dt bias (four pairs, eight genes). In addition, in the no biased pattern (At/Dt = 1 under both CK and UV‐B stress), only 13 homoeologous pairs showed significant changes in the expression of both At‐ and Dt‐ homoeologs under UV‐B stress (Table S8). Thus, the effects of UV‐B stimulation on At‐ and Dt‐ homoeologs at the same structural gene loci in the GSH metabolic pathway tend to more diverse and independently driven.

### 3.5 Potential Regulatory Genes Involved in the GSH Metabolic Pathway Under UV‐B Stress

Through weighted co‐expression network analysis, we found that only 21 structural DEGs in the GSH metabolic pathway could establish a high degree of co‐correlation with regulatory genes, based on which we therefore uncovered 98 regulatory genes that may be involved in the GSH metabolic process. Comparative transcriptome analysis showed that only 96 of these genes were differentially expressed under UV‐B stress, of which 95 were up‐regulated and one was down‐regulated (Table S7). Therefore, these 96 genes were used for the subsequent analyses (Table S7). These regulatory genes can be classified into 13 gene families, the *WRKY* family had the most abundant gene members (24), and the *B3*, *HD‐Zip*, *NF‐X1*, and *RAV* families had the least gene members (2). In addition, from the co‐expression networks, a *WRKY28* gene, *Gohir.D10G039400*, was correlated with the largest number of structural genes (6), but 18 regulatory genes from *bHLH*, *C3H*, *ERF*, *GRAS*, *HSF NAC*, *WRKY* gene families were each co‐expressed with a corresponding structural gene (Table S7).

Twenty‐five homoeologous pairs (50 genes) in the 96 regulatory DEGs were identified (Table S8). The homoeologous pairs belonging to the *B3*, *bHLH*, *C2H2*, *C3H*, *ERF*, *GRAS*, *HSF*, *MYB*, *NAC*, *NF‐X1*, *RAV*, and *WRKY* gene families exhibited consistent up‐regulation of their expressions under UV‐B stress. Furthermore, the patterns of relative expression between At‐ and Dt‐homoeologs of these regulatory genes could be categorized into the unbiased (20 no bias and two lost bias) and biased expression (one At bias, one get At bias, and one get Dt bias) (Table S8). These results show that, in most cases, the effects of UV‐B stress on At‐ and Dt‐homoeologs at each regulatory locus are often unbiased.

### 3.6 qRT‐PCR Verification

**FIGURE 7:**
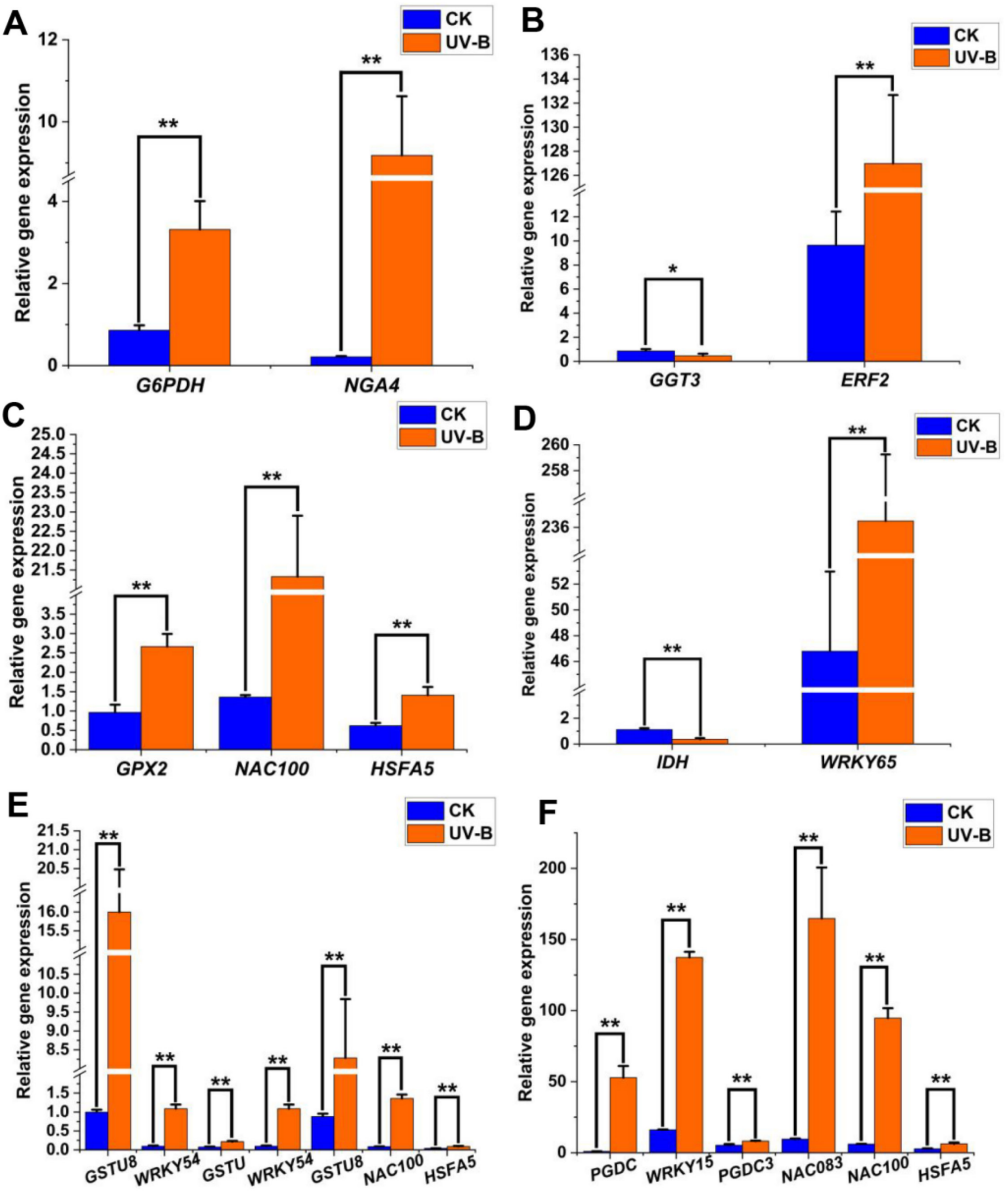
qRT‐PCR analysis of expression changes of representative structural genes and their regulatory genes in the GSH metabolic pathway under UV‐B stress. (A–F) The relative expression of *G6PDH*, *GGT*, *GPX*, *IDH*, *GST*, and *PGDC*, and their regulatory genes. Statistical significance was calculated using *T*‐test; *p < 0.05, **p < 0.01.

To confirm the reliability and validity of the results of RNA‐sequencing, nine structural genes and their potential eight regulatory genes (Table S2) were randomly selected for qRT‐PCR analysis under CK and UV‐B stress conditions. As expected, the qRT‐PCR profiles showed consistent results with RNA‐sequencing, with 15 up‐regulated and only two (*GGT3* and *IDH*) down‐regulated expression under UV‐B stress (Tables S5 and S7). In general, the expression of regulatory genes was more enhanced than that of structural genes. However, the expression levels of a structural genes, *GSTU8*, were higher than their regulatory genes after UV‐B stress (Figure 7).

## 4 Discussion

Previous studies have reported that UV‐B radiation can adversely affect plants and cause oxidative damage (Kakani et al. 2003; Shi and Liu 2021). In this study, we confirmed that after UV‐B treatment (16 kJ m^−2^ d^−1^), the ROS content in the leaves of *G. hirsutum* significantly increased, and the leaf damage subsequently appeared after 2 days (Figure 1). Although flavonoids, glutathione, phenolic compound, terpenoid, and phenylpropanoid (Dias et al. 2020; Xie et al. 2022) all played critical roles in ROS scavenging under UV‐B stress, few studies have focused on the entire metabolic pathway of the antioxidants to investigate their molecular operating mechanisms. Particularly in polyploid plants, such as *G. hirsutum*, two separated and parallel metabolic pathways are combined into a common nucleus through hybridization and whole genome doubling. The evolutionary mechanism of the duplicated pathways is still largely unknown. Here, using the GSH pathway as an example, we investigated the gene composition, expression pattern, and regulatory network of the duplicated pathway during evolution and focused on the response performance of the pathway to UV‐B stress in the tetraploid cotton. This study lays the foundation for better elucidation of the molecular dynamics of specific pathway in polyploid plants.

### 4.1 Evolution of the Structural and Regulatory Genes in the GSH Metabolic Pathway

In this study, we reexamined the evolutionary patterns of the coding genes of 12 key enzymes in the GSH pathway at the genome‐wide level of upland cotton. As a result, we not only found duplicated characteristics of 12 structural enzyme gene families, but also revealed the complex expression patterns of the gene members in each family. Using the largest *GST* family as an example, we identified 121 *GST* members from the cotton genome (UTX_v2.1). Further analyses revealed that these genes were associated with 76 loci, 59% of which showed amplification due to additivity of biparental subgenomic homoeologs. In addition, in our ML tree (Figure 2), the topological clustering of these *GST* genes corresponds exactly to their nine classes of functional annotations (*DHAR*, *EF1Bγ*, *Lambda*, *MGST, Phi*, *Tau*, *TCHQD*, *Theta*, and *Zeta*). This phylogenetic profile indicates that functional redifferentiation may be a significant factor contributing to the expansion of this gene family. Notably, in this study, we newly identified four *MGST* genes (two loci). However, in our ML tree, these genes are not included in the clade where most of the other *GST* genes are placed. In eukaryotes, GSTs can be subdivided into three major protein families based on their cellular localization: cytosolic GSTs, mitochondrial GSTs, and microsomal GSTs. Microsomal GSTs (MGST), also known as MAPEG (membrane‐associated proteins involved in eicosanoid and glutathione metabolism), are integral membrane proteins that are not evolutionarily related to the other major classes (Allocati et al. 2018). Clearly, this evolutionary divergence is well illustrated in our ML tree.

From the expression point of view, apart from the *Ef1bγs* and *TQHODs*, all other seven types (*DHAR*, *Lambda*, *MGST, Phi*, *Tau*, *Theta*, and *Zeta*) of *GST* genes showed differential expression in response to UV‐B stress (Table S3). Furthermore, even within the same type, the expression direction and levels of these *GST* genes are largely different (Table S3). For instance, in the *Tau* genes, we identified 35 up‐regulated and six down‐regulated genes, respectively (Table S3). This situation suggests a complex adaptation‐specific expression of different *GST* genes under UV‐B stress. In addition, the expression levels of the cotton *GST* genes under UV stress were differently altered, ranging from a 1736‐fold up‐regulation (*Gohir.A13G154000*) to a 397‐fold down‐regulation (*Gohir.D04G137100*) (Table S3). As a ubiquitous gene family, the *GST* genes play a crucial role in ROS detoxification, and this differential expression pattern among *GST* gene members under stress conditions is not uncommon in plants. In *Medicago ruthenica*, 66 *GST* genes were identified, of which 14 genes were remarkably affected under drought stress (Wang et al. 2021). In upland cotton, some authors (Li et al. 2019) found significant changes in the expression levels of 17 *GST* genes between resistant and susceptible cultivars under *Verticillium* stress by more than 2.0‐fold. Interestingly, in this study, 6 of the above 17 *GST* genes (*Gohir.A02G027200*, *Gohir.A09G161200*, *Gohir.A09G161400*, *Gohir.A09G161500*, *Gohir.A11G225300*, and *Gohir.D13G156800*) also showed significant changes in expression levels under UV‐B stress. In particular, three genes (*Gohir.A09G161200*, *Gohir.A09G161400*, and *Gohir.A09G161500*) in a gene cluster on chromosome 09 of the upland cotton subgenome A, which have been shown to be involved in *Verticillium* resistance, were substantially up‐regulated by 4–97 fold under UV‐B stress (Table S3). These results indicate that some *GST* genes may have overlapping functions in response to different stresses. Similarly, the gene members in the other 11 structural enzyme families in the GSH metabolic pathway exhibited different expression levels and expression changes (up‐ or down‐regulation) (Figure 6), implying that there is a complex functional assignment and reprogramming among the gene members under UV‐B stress.

Regulatory genes constitute a critical component of the signaling network by controlling the expression of structural genes involved in different defense pathways (Han et al. 2019). Several key regulatory genes such as *WRKY*, *HSF*, and *ERF* have been identified in the GSH metabolic pathway in *A. thaliana* and *Solanum lycopersicum* (Han et al. 2019; Jahan et al. 2019). Here, using the co‐expression networks, we predicted that at least 98 regulatory genes might involve in the cotton *GSH* metabolic pathway, and 96 genes were differential expressed under UV‐B stress, ranging from up‐regulated 2055‐fold (*Gohir.D07G155016*, *MYB14*) to down‐regulated 20‐fold (*Gohir.A09G003800*, *HSPC2*) (Table S7). In addition, we found that among these regulatory genes, the *WRKY* gene family had the highest number of genes (24), followed by *C2H2* (14), *HSF* (11), *ERF* (10), and nine other regulatory gene families (< 10 genes) (Table S7). These regulatory genes are generally very active in regulating ROS homeostasis in response to other biotic and abiotic stresses (Akbar et al. 2020). For example, in sugarcane, the expression of several *WRKY*, *NAC*, and *bHLH* genes were significantly enhanced in resistance to mosaic virus (Akbar et al. 2020; Mijiti et al. 2022). In this study, we found a *WRKY* gene (*Gohir.D10G039400*) was up‐regulated 159‐fold under UV‐B stress. In plants, WRKY is a major transcription factor (TF) family, and is involved in diverse biotic or abiotic stress responses as well as in developmental and physiological processes. In pepper, a *WRKY* gene (*CaWRKY27b*) could not only promote the immune response to the infection of *Ralstonia solanacearum*, but also improve resistance to high‐temperature and high‐humidity stress (Yang et al. 2022). In *A. thaliana*, the homolog of the cotton *WRKY* gene, *Gohir.D10G039400*, displayed significant responses to multiple stresses such as osmotic and cold (Boro et al. 2022). Moreover, it has been found that the up‐regulated expression of other *WRKY* gene*s* in rice have enhanced resistance to UV‐B stress (Wang et al. 2007). In this study, we also found a *NAC* gene (*NAC081*, *Gohir.A05G382000*) was up‐regulated 1187‐fold under UV‐B stress. NAC is considered to be an important regulator of plant tolerance to abiotic stresses by enhancing the ability to scavenge ROS (Mijiti et al. 2022). Overexpressing a *NAC4* gene of *Tamarix hispida* in *Tamarix* and *Arabidopsis* conferred salt and drought stress tolerance to these transgenic plants (Mijiti et al. 2022). These studies show that the regulatory network of the GSH pathway under UV‐B stress may overlap with that under other stresses to some extent.

### 4.2 Homoeolog Evolution in the GSH Metabolic Pathway of *G. hirsutum*

Allopolyploidization is the integration of heterologous subgenomes into a common nucleus (Wendel, Flagel, and Adams 2012). After polyploidization, subgenomic homoeologs usually do not evolve in parallel, and subgenome dominance or asymmetry is prevalent in allopolyploids (Chaudhary et al. 2009). Furthermore, from the point of view of the gene locus, unequal contribution by the At‐ and Dt‐homoeologs to the cotton transcript pool of any single gene is common phenomena (Flagel and Wendel 2010). In this study, we revealed that 64% (80% and 26.5%) (26) of structural and regulatory gene loci (Tables S3 and S5) were duplicated by retaining both At‐ and Dt‐homoeologs in the GSH metabolic pathway of *G. hirsutum*. However, the relative expression patterns of the doubled homoeologs at structural and regulatory gene loci are remarkably different.

In the case of structural genes of the GSH metabolic pathway, there is a general imbalance or biased evolution between At‐ and Dt‐homoeologs at the expression level of each locus. A total of 72 structural homoeologous pairs were retrieved from cotton leaf transcriptomes under CK and UV‐B stress (SRR22018192‐SRR22018197) (Table S8). Of the 72 homoeologous pairs, only 12 structural pairs (*Gohir.A09G192401* vs. *Gohir.D09G186600, Gohir.A06G107600* vs. *Gohir.D06G109900, Gohir.A06G069200* vs. *Gohir.D06G067900, Gohir.A04G097000* vs. *Gohir.D04G136800, Gohir.A04G097300* vs. *Gohir.D04G137100, Gohir.A08G037800* vs. *Gohir.D08G048100, Gohir.A08G181700* vs. *Gohir.D08G200300, Gohir.A10G068200* vs. *Gohir.D10G083000, Gohir.A11G157300* vs. *Gohir.D11G164300, Gohir.A12G045000* vs. *Gohir.D12G043800, Gohir.A13G152500* vs. *Gohir.D13G156900*, and *Gohir.A07G027000* vs. *Gohir.D07G031000*) maintained the same relative expression ratios of At‐ to Dt‐homoeologs under UV‐B stress (Table S8). This result suggests that in most cases, UV‐B stimulation fail to have a concerted effect on At‐ and Dt‐ homoeologs at each structural locus. In other words, At‐ and Dt‐ homoeologs at most structural loci respond to UV‐B stress independently of each other. Meanwhile, we also found that 10 homoeologous pairs from two structural gene families (seven *GSTs* and three *GPXs*) showed biased expression patterns (get At bias and get Dt bias) only under UV‐B stress, suggesting an expression repartitioning between biparental homoeologs during UV‐B defense.

In detail, we revealed that the expression change in the ratio of At‐ to Dt‐homoeologs of each structural gene under UV‐B stress was inconsistent compared with the CK condition, ranging from a 230‐fold increase in *GSTU8* (*Gohir.A09G161200* vs. *Gohir.D09G156900*) to a 27‐fold decrease in *GGT1* (*Gohir.A11G053200* vs. *Gohir.D11G056700*) (Table S8). The *GSTUs*, belonging to a plant‐specific subfamily of the *GST* gene family, are important structural genes in several metabolic pathways, and encode a common class of detoxifying and ROS‐scavenging enzymes (Herrera‐Vasquez et al. 2021). In *A. thaliana*, 17 *GSTU* genes were found to be up‐regulated under UV‐B stress, and 9 of them were positively regulated by transcription factors TGA2/5/6 (Herrera‐Vasquez et al. 2021). In this study, we identified 41 *GSTU* genes that were differentially expressed under UV‐B stress (Table S3). These 41 *GSTUs* included 10 homoeologous pairs (10 gene loci) (Table S3). Previous studies displayed that the *GSTU* genes were always up‐regulated under stresses. Over‐expression of a *AtGSTU19* provided obvious tolerance to salt, drought and methyl viologen stresses in *Arabidopsis* (Xu et al. 2016). Here, under UV‐B stress, we also found six down‐regulated *GSTUs* (Table S3). Notably, although both At‐ and Dt‐ homoeologs of the 10 cotton *GSTU* loci had consistent up‐ or down‐regulated expression trends, the relative expression ratios of the two homoeologs were not consistent under UV‐B stress, with a variety of relative expression patterns such as no bias, loss bias, At bias, and Get At bias (Table S8). Therefore, the At‐ or Dt‐ homoeologs of these *GSTUs* may play different roles to defense excess UV‐B radiation independently. Further functional validation should be carried out in the future.

Previous studies have confirmed that in polyploid cottons, both homoeologs of some structural genes can also exhibit different expression in an environmental adaptation manner (Liu and Adams 2007; Flagel and Wendel 2010; Bao et al. 2019; Peng et al. 2022). For example, there is a cotton alcohol dehydrogenase gene, *AdhA*, whose At‐ and Dt‐homoeolog show different expression levels under water‐submersion, cold stress, and dark stress (Liu and Adams 2007). Here, we also identified 19 loci where only a single At‐ or Dt‐ homoeolog was independently differentially expressed under UV‐B stress (Table S8). Flagel et al. (2009) have suggested that expression divergence between homoeologs may be controlled by large‐scale linkage in some genomic regions or small‐scale fine regulation (Flagel et al. 2009). Sequence divergences in the promoters or other non‐codon regions between two hetero‐homoeologs of each structural gene locus in *G. hirsutum* provide more opportunities for regulatory changes (Chaudhary et al. 2009; Flagel et al. 2009; Bao et al. 2019). Thereafter, this inconsistent expression between duplicated homoeologs of certain structural loci may trigger further partitioning and redefinition of their functions, which in turn promotes the retention of those duplicated homoeologs during subsequent evolutionary processes (Liu and Adams 2007).

In contrast, among the regulatory genes of the GSH metabolic pathway, the relative expression between At‐ and Dt‐homoeologs at each locus tends to follow an unbiased pattern. Among 25 homoeologs pairs, 21 pairs (20 no bias, 1 A bias) maintained the same relative expression change of At‐ to Dt‐ homoeolog under both CK and UV‐B stress conditions (Table S8). Given that both homoeologs of these regulatory genes are up‐regulated in response to UV‐B stress, this unbiased pattern of relative homoeolog expression suggests that UV‐B stimulation acts equally on both homoeologs of each regulatory locus. Some authors used the gene balance hypothesis to explain that dose‐sensitive genes (e.g., regulatory genes) are more likely to be retained as duplicates (Birchler and Veitia 2007). Similar results have been found in polyploid wheat, where homoeologous copies of regulatory genes not only had highly similar co‐expression patterns, but were also more likely to be retained than other genes (Evans, Arunkumar, and Borrill 2022).

In summary, we herein reveal the complex evolution of a duplicated GSH metabolic pathway in the allopolyploid *G. hirsutum*, which, rather than combining two diploid pathways in parallel in a common nucleus, influences the expression of structural homoeologs through a reticulated and cross‐subgenomic regulatory system, and thereby increases the correlation and integrality of the duplicated pathway. Our present study not only explored the duplicated structural gene framework of the GSH metabolic pathway of *G. hirsutum* at whole genome‐wide level, but also further predicted several potential reticulated regulatory systems in the pathway under UV‐B stress. This study broadens our understanding of the molecular dynamics underlying the UV‐B response in *G. hirsutum* and polyploid plants in general.

## Author Contributions

**Xiaolin Song:** formal analysis (lead), writing – original draft (lead), writing – review and editing (lead). **Xiaoyu Yin:** formal analysis (supporting), writing – original draft (supporting), writing – review and editing (supporting). **Yingjie Zhu:** formal analysis (supporting), resources (lead). **Qi Su:** writing – original draft (supporting), writing – review and editing (supporting). **Ying Bao:** project administration (lead), writing – original draft (equal), writing – review and editing (equal).

## Conflicts of Interest

The authors declare no conflicts of interest.

## Supporting information




**Table S1** Transcriptome information used in this study.

**Table S2** Primers information for qRT‐PCR analysis.

**Table S3** Structural genes involved in the glutathione (GSH) metabolism of *Gossypium hirsutum* under UV‐B stress.

**Table S4** Protein sequence similarity of the genes in the glutathione (GSH) pathway in *Gossypium hirsutum*.

**Table S5** Genes involved in the glutathione (GSH) metabolic pathway of *Gossypium hirsutum*.

**Table S6** Assembly information in leaves transcriptomes of *Gossypium hirsutum* under UV‐B stress.

**Table S7** Differentially expressed genes (DEGs) in the glutathione (GSH) metabolic pathway of *Gossypium hirsutum* under UV‐B stress.

**Table S8** Homoeologous gene pairs involved in the GSH metabolic pathway of *Gossypium hirsutum* under UV‐B stress.

## Data Availability

All relevant figures are within the manuscript and its Supporting Information. The raw data and code used for the qRT‐PCR and WGCNA analysis deposited in Dryad (http://datadryad.org/stash/share/39vfQJ9hqnMiMVukzk‐9N5zL8EQl8DTuPmeEKJvdyqo. doi:10.5061/dryad.zgmsbccnd).
